# Impact of Metal Nanoform Colloidal Solution on the Adaptive Potential of Plants

**DOI:** 10.1186/s11671-016-1294-z

**Published:** 2016-02-15

**Authors:** Nataliya Taran, Ludmila Batsmanova, Mariia Kovalenko, Alexander Okanenko

**Affiliations:** Institute of Biology, Taras Shevchenko National University of Kyiv, 64/13, Volodymyrska Street, Kyiv, 01601 Ukraine

**Keywords:** Plants, Nanoparticles, Catalase, Superoxide dismutase, Pigments, Lipid peroxidation, Factor of antioxidant activity

## Abstract

Nanoparticles are a known cause of oxidative stress and so induce antistress action. The latter property was the purpose of our study. The effect of two concentrations (120 and 240 mg/l) of nanoform biogenic metal (Ag, Cu, Fe, Zn, Mn) colloidal solution on antioxidant enzymes, superoxide dismutase and catalase; the level of the factor of the antioxidant state; and the content of thiobarbituric acid reactive substances (TBARSs) of soybean plant in terms of field experience were studied. It was found that the oxidative processes developed a metal nanoparticle pre-sowing seed treatment variant at a concentration of 120 mg/l, as evidenced by the increase in the content of TBARS in photosynthetic tissues by 12 %. Pre-sowing treatment in a double concentration (240 mg/l) resulted in a decrease in oxidative processes (19 %), and pre-sowing treatment combined with vegetative treatment also contributed to the reduction of TBARS (10 %). Increased activity of superoxide dismutase (SOD) was observed in a variant by increasing the content of TBARS; SOD activity was at the control level in two other variants. Catalase activity decreased in all variants. The factor of antioxidant activity was highest (0.3) in a variant with nanoparticle double treatment (pre-sowing and vegetative) at a concentration of 120 mg/l. Thus, the studied nanometal colloidal solution when used in small doses, in a certain time interval, can be considered as a low-level stress factor which according to hormesis principle promoted adaptive response reaction.

## Background

Abiotic stress is produced by a series of factors like extreme temperatures, chemical compounds, water deficit, and an excess of heavy metals. Metabolic processes in plants continually produce reactive oxygen species (ROS) in structures like chloroplasts, mitochondria, peroxisomes, the endoplasmic reticulum (ER), and plasma membranes [1]. ROS is controlled by various enzymatic and non-enzymatic antioxidative systems. Enzymatic antioxidants include catalase (CAT), ascorbate peroxidase (APX), guaiacol peroxidase (GPX), superoxide dismutase (SOD), and enzymes that detoxify lipid peroxidation (LP) products, and non-enzymatic antioxidants include ascorbic acid (AA), glutathione (GSH), tocopherols (TOCs), carotenoids (CARs), and phenolic compounds. In addition, an array of enzymes, such as monodehydroascorbate reductase (MDHAR), dehydroascorbate reductase (DHAR), and glutathione reductase (GR), is needed for the regeneration of the active forms of the antioxidants.

The unique properties of nanomaterials and nanoclusters formed are widely used in various fields of activity now [2, 3]. The metastable unbalanced state of nanoparticles does not allow the prediction of their influence upon the physiological and biochemical processes taking place in plants; therefore, the very urgent problem of identifying the degree of stress impact in a wide range of concentrations is taking place today. There is no single opinion on the impact of nanoparticles upon physiological and biochemical processes in the literature available—both positive and negative effects are noted. The pro-oxidant effect of synthesized fullerene-containing (10–6 mol/l by C60) supramolecular composites on the lipid peroxidation processes has been revealed [4, 5].

It is known that nanoparticles have positive morphological effects like the enhancement of seed germination rates and the improvement of root and shoot formation and their ratio, as well as the accumulation of vegetative biomass of seedlings in many crop plants [6]. Nanoparticles’ influence on the cell level thus increases the pace of physiological processes in plants. The nanoparticles of zinc, cuprum, iron, etc. revealed by now are up to 40 times less toxic than the salts [7, 8]. They are gradually absorbed while their ionic forms are immediately included into the biochemical reactions. Nanoparticles take part in the electron transfer in plants and thus increase the activity of plant enzymes, intensify photosynthesis processes, and have a direct influence on the plant mineral nutrition [9–11]. The colloidal solutions containing biologically active metals are now being widely used along with traditional biological preparations. There are preliminary conclusions about the positive effects of these preparations on the productivity and plant resistance to adverse environmental factors [12]. It was found also that various nanoparticles exhibit antioxidant enzyme-like ability: *n*CeO_2_ and *n*Fe_3_O_4_ reveal catalase activity; *n*CeO_2_, *n*Fe_3_O_4_, *n*Co_3_O_4_, *n*MnO_2_, *n*CuO, and *n*Au exhibit peroxidase activity; and *n*CeO_2_, *n*Pt, and fullerene demonstrate superoxide dismutase property [13]. There is an opinion that the presence of nonpolar environments seems to enhance the reactivity of the fullerene molecule toward OH radicals compared to the gas phase. Energetic considerations show that, once a first radical is attached to the fullerene cage, further additions are increasingly feasible, suggesting that fullerene can act as OH radical sponges [14]. Unfortunately, it is next to impossible to separate these activities in experiments: *n*TiO_2_-A enhanced the activities of SOD, CAT, APX, and GPX in spinach [15] and GPX, SOD, and CAT in *Lemna minor* [16]. It is known also about the photoreduction activity of *Vigna radiata* isolated chloroplasts exposed to *n*Mn. Nanoparticles are revealed to modulate the activity of photosystem II (PSII) by enhancing the splitting of water and oxygen evolution thus improving the photophosphorylation activity of the electron transport chain. The enhanced activity of the CP43 protein of a PSII Mn_4_Ca complex influenced better phosphorylation in the electron transport chain in a *n*Mn-treated chloroplast [17].

Thus, the role of nanoparticles’ chemical attributes on the modulation of the antioxidant defense system in plants is not clear and a topic worthy of attention. Based on this information, the purpose of our research was to study the effect of different concentrations (120 and 240 mg/l) of trace element (Ag, Cu, Fe, Zn, Mn) nanoform colloidal solution as biogenic minerals used in agricultural technologies for plant nutrition, regulation of physiological processes, and the development of plant adaptive reactions in a field experiment.

## Methods

The object of the study was soybean plants of the early ripening variety (Annushka—Ukrainian selection, PC “Soeviy Vik”). The investigation was performed in field conditions. The soil of the experimental field was typical black soil with humus content in the arable soil layer equal to 4.38–4.53 %, pH of salt extract—6.9–7.3, nitrogen—0.27–0.31 %, phosphorus—0.15–0.25 %, and potassium—2.3–2.5 %. The farming equipment is common to the northern forest steppe of Ukraine. The treatment of seeds and plants with a colloidal solution of metal nanoparticles was performed under the following scheme: 1—control, treatment with water; 2—pre-sowing seed treatment with complex metal colloids at a concentration of 120 mg/l; 3—pre-sowing seed treatment with complex metal colloids at a concentration of 240 mg/l; and 4—pre-sowing seed treatment combined with vegetative treatment (spraying in the budding stage) with complex metal colloids at a concentration of 120 mg/l. Analysis of control and treated soybean plants was carried out at the stage of three true leaves and at vegetation after treatment with nanoparticle solutions.

Processing of the engineering nanoform metal colloidal solution and study of its physical properties were determined at the National University of Life and Environmental Sciences of Ukraine. One of the most promising methods of nanomaterial production was used—the spark erosion treatment of the material in water and formation of colloidal solutions with nanoparticles. A specially developed pulse power source was used to initiate the discharge between granules (copper, silver, iron, aluminum, zinc, graphite, etc.) dipped into the deionized water. The combination of intense heat and force action on the material during ultra-short time intervals in such a discharge gives the possibility to obtain nanoparticles with a non-equilibrium structure, increased level of free energy, and sizes of 20–100 nm (Table 1). The nanoparticles derived from this technology are a covered nanohydration shell that is easily replaced by a sheath of biocompatible organic molecules [18]. The concentration of metal nanoparticle complex (Fe, Mn, Mo, Co, Cu, Zn, Ag) is 120 mg of metal per liter H_2_O.

**Table 1:** Characteristics of the colloidal solution of metal nanoparticles [18]

Metal	Concentration, mg/l	The average diameter, nm	Phase composition
Ag	150	30–50	Ag, Ag_2_O
Cu	200	100–150	Cu, CuO, Cu_2_O
Fe	300	20–30	Fe, Fe_2_O_3_, Fe_3_O_4_
Zn	150	30–50	Zn, ZnO
Mn	150	20–30	Mn, Mn_3_O_4_, Mn_2_O_7_

### Enzymatic Activity

The photosynthetic tissue of soybean plants (300 mg) were washed with distilled water and homogenized with 1.5 ml of 50 mM phosphate buffer (pH 7.8). The homogenate was centrifuged for 15 min at 12 000 rpm at 4 °C. The supernatant was stored at 4 °C and used to determine enzyme activity and protein content.

The activity of SOD (EC 1.15.1.1) was determined according to the ability to inhibit reduction of nitroblue tetrazolium (NBT) at *λ* = 560 nm. The unit of enzyme activity was 50 % inhibition of formazan formation. SOD activity is expressed in arbitrary units per milligram protein [19].

The activity of CAT (EC 1.11.1.6) was determined according to Aeby [20]. Activity was expressed as arbitrary units per milligram protein.

### Determining the Level of LP

The level of lipid peroxidation was evaluated by the accumulation of thiobarbituric acid reactive substances (TBARSs) according to Kumar and Knowles [21] with modifications. The intensity of lipid peroxidation (LP) was evaluated by the content of malonic dialdehyde that was determined by 2-thiobarbituric acid reaction. Photosynthetic tissues (200 mg) were homogenized with a small amount (1.5 ml) of Tris-NaCl buffer (pH 7.6), and the volume of the extract was adjusted to 3 ml. To the resulting homogenate, 1 ml of 0.5 % solution thiobarbituric acid (TBA) and 2 ml of 20 % trichloroacetic acid (TCAA) were added. Test tubes with homogenates withstand 30 min in a boiling water bath, followed by centrifugation at 3000 rpm for 10 min. Light absorption was recorded at *λ* = 533 nm. Content of TBARS was expressed in micromoles of malondialdehyde using a molar extinction coefficient 1.55 × 105 cm^−1^ M^−1^.

The integral indicator of overall antioxidant activity was calculated by formula 1.

1 $$ F=\left({\mathrm{CAT}}_{\mathrm{a}}\times {\mathrm{SOD}}_{\mathrm{a}}\right)/{\mathrm{TBARS}}_{\mathrm{con}} $$

where *F*—factor antioxidant status, CAT_a_—CAT activity, SOD_a_—SOD activity, and TBARS_con_—TBARS content [22] characterizing antioxidant potential.

The determination of dry biomass was performed according to GOST 16932-93. The determination of protein was performed based on the method of Bradford [23]. Statistical analysis of the data obtained was performed with the help of program Statistica 6.0. The differences are considered significant at a value *P* ≤ 0.05.

## Results and Discussion

**Fig. 1:**
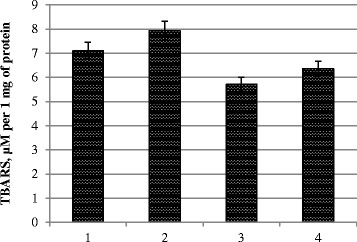
The content of TBARS in photosynthetic tissues. *1* control, treatment with water; *2* pre-sowing seed treatment with complex metal colloids at a concentration of 120 mg/l; *3* pre-sowing seed treatment with complex metal colloids at a concentration of 240 mg/l; *4* pre-sowing seed treatment combined with vegetative treatment (spraying in the budding stage) with complex metal colloids at concentration 120 mg/l

Analysis of the results shows that oxidative processes develop a variant of pre-sowing seed treatment with metal nanoparticle colloidal solution at a concentration of 120 mg/l, as evidenced by the increase (12 %) of the content of TBARS in photosynthetic tissues (Fig. 1). The pre-sowing treatment with a double concentration of the solution (240 mg/l) resulted in a decrease of oxidative processes (19 %), and the pre-sowing treatment combined with vegetative treatment also contributed to the reduction of TBARS (by 10 %).

**Fig. 2:**
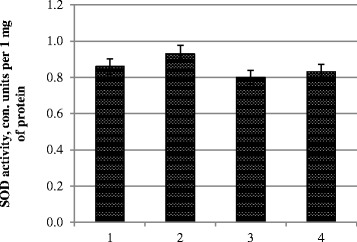
SOD activity in photosynthetic tissues. *1* control, treatment with water; *2* pre-sowing seed treatment with complex metal colloids at a concentration of 120 mg/l; *3* pre-sowing seed treatment with complex metal colloids at a concentration of 240 mg/l; *4* pre-sowing seed treatment combined with vegetative treatment (spraying in the budding stage) with complex metal colloids at a concentration of 120 mg/l

The variant with an increasing TBARS content observed an increased activity of SOD; two other variants have SOD activity at the control level (Fig. 2).

**Fig. 3:**
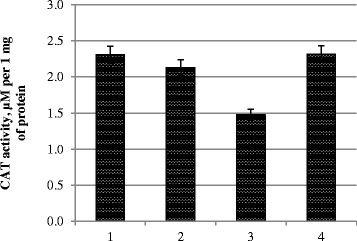
CAT activity in photosynthetic tissues. *1* control, treatment with water; *2* pre-sowing seed treatment with complex metal colloids at a concentration of 120 mg/l; *3* pre-sowing seed treatment with complex metal colloids at a concentration of 240 mg/l; *4* pre-sowing seed treatment combined with vegetative treatment (spraying in the budding stage) with complex metal colloids at a concentration of 120 mg/l

**Fig. 4:**
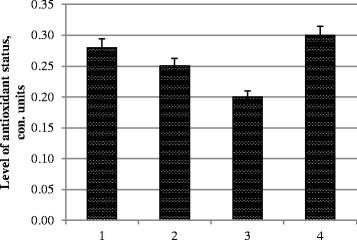
Level of antioxidant status (in arbitrary units)—the ratio of antioxidant to pro-oxidant. *1* control, treatment with water; *2* pre-sowing seed treatment with complex metal colloids at a concentration of 120 mg/l; *3* pre-sowing seed treatment with complex metal colloids at a concentration of 240 mg/l; *4* pre-sowing seed treatment combined with vegetative treatment (spraying in the budding stage) with complex metal colloids at a concentration of 120 mg/l

CAT activity decreased in all variants (Fig. 3). In characterizing the adaptive responses of plants, the most revealing factor is the level of antioxidant status, which takes into account the ratio of antioxidant to pro-oxidant. The most balanced index was in variant 4 (dual treatment of plants with metal nanoparticle complex at a concentration of 120 mg/l)—it exceeded the control variant here (Fig. 4).

Thus, the most “antistress” treatment judging upon TBARS was variant 3—pre-sowing seed treatment with complex metal colloids at a concentration of 120 mg/l, whereas in variant 2 the content of TBARS overcame control despite maximal SOD activity. On the other side, taking into account all indexes, the most successful combination was applied in variant 4—pre-sowing seed treatment combined with vegetative treatment (spraying in the budding stage) with complex metal colloids at a concentration of 120 mg/l. It is worthy to note that chlorophyll decrease did not take place here.

Data available in the literature do not contradict ours. Significant reduction of H_2_O_2_ accumulation and lipid peroxidation (TBARS content decreases, same as the index of ROS production) at 25 and 50 parts per million (ppm, mg/l, or mg/kg) of *n*Ag-treated (25–400 ppm) 7-day-old *Brassica juncea* seedlings was observed. Improved photosynthetic quantum efficiency and higher chlorophyll content were recorded in leaves of treated seedlings, whereas levels of TBARS and H_2_O_2_ decreased herein. Nanoparticle treatment induced the activities of specific antioxidant enzymes like GPX, CAT, and APX, causing the ROS level to decrease. Besides an increase in chlorophyll *a* content, PSII quantum efficiency (Fv/Fm) was observed at 25, 50, and 100 ppm *n*Ag treatment. The higher quantum efficiency (Fv/Fm) shows that the greater number of reaction centers are “open” to hold a light reaction that reduces the probability of the ROS generation. It was suggested that *n*Ag increased the efficiency of redox reactions, based on the ability of *n*Ag to act as an electron transfer center increasing the efficiency of redox reactions [24].

**Fig. 5:**
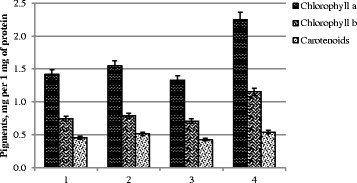
The content of photosynthetic pigments. *1* control, treatment with water; *2* pre-sowing seed treatment with complex metal colloids at a concentration of 120 mg/l; *3* pre-sowing seed treatment with complex metal colloids at a concentration of 240 mg/l; *4* pre-sowing seed treatment combined with vegetative treatment (spraying in the budding stage) with complex metal colloids at a concentration of 120 mg/l

There are more reports dealing with the stimulating effect of metal nanoparticles upon the chlorophyll content in the literature. Silver nanoparticle treatment of the common bean (*Phaseolus vulgaris*) and corn (*Zea mays*) in a concentration of 60 ppm induced a significant increase of chlorophyll *a*, *b*, and carotenoids compared to control. But pigment content began to decrease in both species at a higher concentration [25]. Soybean treated with iron oxide nanoparticles showed a chlorophyll-level increment without a trace of toxicity. It was concluded that they may have influence on both the biochemical and enzymatic efficiency of various reactions of photosynthesis [26]. In another investigation, *Z. mays* seeds germinated in magnetic fluid (ferrophase content was 2.03 × 10^17^ particles within 1 ml) resulted in a chlorophyll (*a*, *b*) and carotenoid content increase (of about 30 %) for a fraction of magnetic fluid of 100–200 μl/l with a diminution at 300 μl/l almost to the control level in young plants [27]. Our study showed only a slight increase of chlorophyll content in the variant with pre-sowing treatment combined with a vegetative one. It corresponds to the highest antioxidant status here. The content of carotenoids was a bit lower compared to the control in all variants of the experiment especially in variant 3 (Fig. 5).

**Fig. 6:**
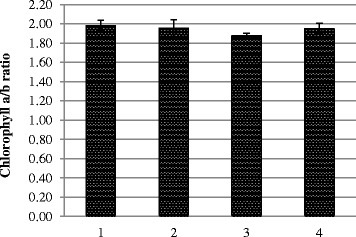
Chlorophyll *a*/*b* ratio. *1* control, treatment with water; *2* pre-sowing treatment with complex metal colloids at a concentration of 120 mg/l; *3* pre-sowing treatment with complex metal colloids at a concentration of 240 mg/l; *4* pre-sowing treatment combined with vegetative treatment (spraying in the budding stage) with complex metal colloids at a concentration of 120 mg/l

It is well known that chlorophyll ratio (chlorophyll *a*/chlorophyll *b*) is the best indicator which provides indirect information on the activity of the light-harvesting complex II (LHCII) from the PSII. It was lowest (Fig. 6) in variant 3 on the background of a general reduction of chlorophyll content mainly due to the falling number of chlorophyll *a*. It could be considered as a possible damage result because of toxicity of the high nanoparticle dose.

Both forms of chlorophyll are involved in light harvesting, whereas special forms of only chlorophyll *a* are linked into energy-processing centers of photosystems. In strong light, photons are abundant, consistent with a substantial capacity for energy processing by leaves (hence the higher chlorophyll *a*/*b* ratio). In weak light, optimization of leaf function calls for greater investment of leaf resources in light harvesting rather than energy processing. Because of how a photosystem looks like (chlorophyll *a* in the middle, chlorophyll *b* at the periphery), the plant can only increase the amount of chlorophyll *b* (there is no way to throw some molecules in the middle of the photosystem). So as it accumulates more chlorophyll *b*, the ratio drops. The chlorophyll *a*/*b* ratio can be a useful indicator of plant state, because this ratio should be positively correlated with the ratio of PSII cores to LHCII. It contains the majority of chlorophyll *b* and has therefore a lower chlorophyll *a*/*b* ratio than other chlorophyll-binding proteins of PSII [28]. Therefore, chlorophyll *a*/*b* ratio can be applied for the rapid detection of plant stress in arid ecosystems [29].

Concerning stress action, it is known that any stress causes oxidative stress followed by damage of the photosynthetic apparatus. For example, heavy metals (Pb, Cu, Cd, Hg) decreased the total chlorophyll content in the leaves of bean seedlings progressively, but the chlorophyll *a*/*b* ratio increased slightly with increasing concentrations of heavy metal [30]. The strong decrease of chlorophyll content was associated with a twofold increase of the chlorophyll *a*/*b* ratio in salt-stressed spinach leaves [31]. Some types of stress increase the ratio of chlorophyll *a* to chlorophyll *b*; other stresses reduce it [32]. Our data allow us to conclude that in variants 2 and 4 plant photosynthesis was affected to a lesser extent.

Carotenoids also participate in photosynthetic energy transduction as an “accessory” to primary pigments (chlorophylls) and perform several functions in photosynthetic membranes. The most important is preventing the formation of singlet oxygen and protecting chlorophylls by quenching their triplet states. Carotenoids play also a central role for chlorophyll-binding proteins of both the antenna system and the reaction center and for D1 protein assembly during its turnover while forming functional PSII complexes. Inhibitors of carotene biosynthesis led to the loss of both PSII activity and D1 protein, indicating the requirement of *β*-carotene synthesis for the reassembly of PSII [33, 34]. Thus, carotenoid content changes in our experiment evidence in favor of variant 4 being optimal for plants.

## Conclusions

Thus, the nanoparticle effect depends upon the plant species, type of nanoparticles, dose of treatment, and method of processing—double treatment at different times (pre-sowing seed treatment as “preparing” and spraying in the budding stage as “fixating” one) caused the best effect. The situation could be considered as hardening. The metal nanoparticles are likely to mobilize hormesis and sensitization through indirect effects on specific biological adaptive mechanisms. Colloidal solution nanometals in small doses used with a certain time interval can be considered as a low-level stress factor that stimulates allostatic response, causing nonlinear modulator changes of endogenously strengthened processes, contributing to the development of adaptive responses and answers.
